# Bacterial Communities and Enzymatic Activities in Sediments of Long-Term Fish and Crab Aquaculture Ponds

**DOI:** 10.3390/microorganisms9030501

**Published:** 2021-02-26

**Authors:** Zhimin Zhang, Qinghui Deng, Lingling Wan, Xiuyun Cao, Yiyong Zhou, Chunlei Song

**Affiliations:** 1 Key Laboratory of Algal Biology, State Key Laboratory of Freshwater Ecology and Biotechnology, Institute of Hydrobiology, Chinese Academy of Sciences, Wuhan 430072, China; zhangzm@ihb.ac.cn (Z.Z.); dengqinghui@ihb.ac.cn (Q.D.); wanlingling@ihb.ac.cn (L.W.); caoxy@ihb.ac.cn (X.C.); zhouyy@ihb.ac.cn (Y.Z.); 2 University of Chinese Academy of Sciences, Beijing 100039, China

**Keywords:** aquaculture practices, pond sediments, bacterial community, hydrolytic enzymes

## Abstract

Aquaculture is among the most important and fastest growing agriculture sectors worldwide; however, it generates environmental impacts by introducing nutrient accumulations in ponds, which are possibly different and further result in complex biological processes in the sediments based on diverse farming practices. In this study, we investigated the effects of long-term farming practices of representative aquatic animals dominated by grass carp (GC, *Ctenopharyngodon idella*) or Chinese mitten crab (CMC, *Eriocheir sinensis*) on the bacterial community and enzyme activity of sediments from more than 15 years of aquaculture ponds, and the differences associated with sediment properties were explored in the two farming practices. Compared to CMC ponds, GC ponds had lower contents of TC, TN, and TP in sediments, and similar trends for sediment pH and moisture content. Sediment bacterial communities were significantly different between GC and CMC ponds, with higher bacterial richness and diversity in GC ponds. The bacterial communities among the pond sediments were closely associated with sediment pH, TC, and TN. Additionally, the results showed profoundly lower activities of β-1,4-glucosidase, leucine aminopeptidase, and phosphatase in the sediments of GC ponds than CMC ponds. Pearson’s correlation analysis further revealed strong positive correlations between the hydrolytic enzyme activities and nutrient concentrations among the aquaculture ponds, indicating microbial enzyme regulation response to sediment nutrient dynamics. Our study herein reveals that farming practices of fish and crab differently affect bacterial communities and enzymatic activities in pond sediments, suggesting nutrient-driven sediment biological processes in aquaculture ponds for different farming practices.

## 1. Introduction

Global aquaculture production has greatly increased with the increase in consumption demand for animal protein and the decline in capture fisheries in recent decades due to increasing populations, and nowadays, it represents a major global agriculture industry sector [1]. The ever-expanding aquaculture is closely associated with the development and application of commercial feeds; however, this leads to heavy nutrient loading and sediment deposition in aquaculture ecosystems [2,3]. At present, aquaculture pond systems function as net nutrient pools [4,5]. Many studies have carried out nutrient budgets in aquaculture systems, revealing that commercial feeds are the primary source of carbon (C), nitrogen (N), and phosphorus (P) in ponds, such as with <17% being assimilated by shrimp [6,7], and 15% of total N and <3% P inputs by harvested fish [5]. Therefore, large amounts of nutrients introduced to ponds end up settling on the pond bottom [8,9]. Moreover, it has been reported that nutrient accumulations of pond sediments increase with total nutrient input [5]. Undeniably, anthropogenic activities and aquaculture practices can deeply affect the strength of nutrient sinks.

Bacteria are responsible for the degradation and recycling of essential elements, such as C, N, and P in sediments. Some studies have revealed that bacterial communities in aquatic environments are strongly influenced by site-specific conditions [10,11]. Nutrient input from farming practices might alter sediment properties, and eventually affect bacterial communities important in regulating the aquatic environment and sediment functioning. Despite the well-known importance of bacterial communities, how the communities in aquaculture ponds assemble in response to long-term aquaculture practices remains rarely understood. If aquaculture primarily affects patterns in bacterial communities of pond sediments, then it could be expected that species-driven farming practices differently drive bacterial community assembly. Moreover, sediments from the same cultured species ponds are likely to share more similar bacterial communities in long-term aquaculture practices. The hypotheses on aquaculture practices in determining patterns in bacterial communities can be only validated through further studies that systematically collect sediment samples from representative ponds.

Bacterial community structure and abundance in tropical marine ecosystems have been used for investigating the impact of fish farms and have proved to be useful as indicators of fish farm footprints [12,13]. Recently, studies have focused on bacterial communities in aquatic farming ponds [14,15]. Zhang et al. (2019) further attempted to detail the ecology of nutrient cycling processes and revealed different communities in shrimp sediments from traditional and higher-place ponds [16], potentially suggesting biological differences in the sediments [17]. Particularly, potential enzymatic activities of bacteria in terrestrial environments have been considered as a feasible method to deduce nutrient limitation steps for environmental microorganisms [18]. However, it is less reported for contrasting aquatic ecosystems, such as static lakes and ponds. Moreover, pond sediments are in extremely anoxic conditions caused by degradations and accumulations of numerous aquaculture wastes. Fish is the most popular cultured species, followed by shellfish. In aquaculture, fish ponds are usually equipped with aerators, but not always in shellfish ponds, such as crab and crayfish ponds, and they have significant differences in farming practices, including feed input and utilization, and culture cycle. For a long-term aquaculture period, species-based aquaculture pond bottom can accumulate amounts of sediments, and nutrients may differ among different species ponds. In this context, however, there remains a gap in our knowledge of the comprehensive sediment ecology of heavily distributed ponds for different farming practices. In the present study, we explored sediment bacterial communities and enzyme activities in fish and crab ponds, as well as their relationships with sediment properties. This valuable information is a fundamental step in understanding biogeochemical processes in aquaculture ponds and evaluating the associated environmental impacts of farming practices.

## 2. Materials and Methods

### 2.1. Experimental Design and Sampling Produce

**Figure 1:**
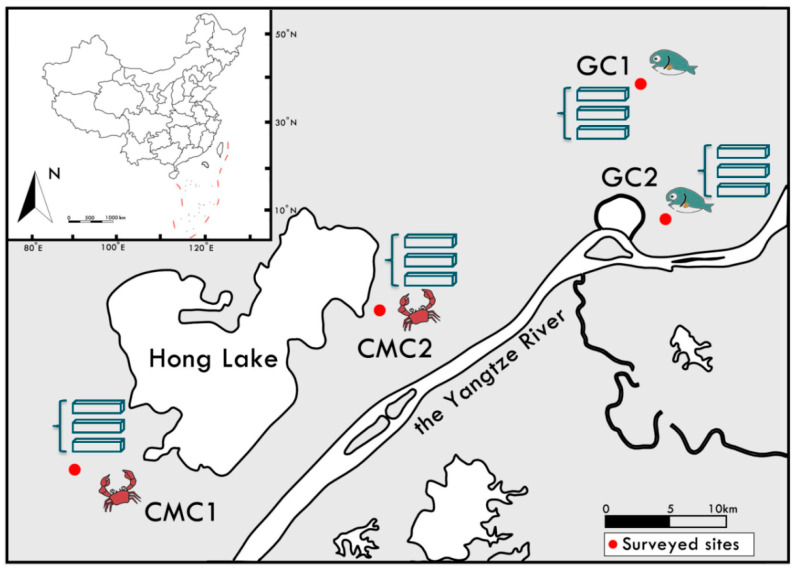
The location of grass carp (GC) and Chinese mitten crab (CMC) ponds sampled around Hong Lake, middle Yangtze River Basin, China. GC1, Grass carp farm 1; GC2, Grass carp farm 2; CMC1, Chinese mitten crab farm 1; CMC2, Chinese mitten crab farm 2. Three ponds were used for sediment collections in each surveyed site.

In this study, two representative aquatic intensive earthen ponds for fish dominated by grass carp (*Ctenopharyngodon idella*) and Chinese mitten crab (*Eriocheir sinensis*) were sampled for sediment collections. They were named GC ponds and CMC ponds with significantly different environmental conditions (Table S1). During the culture cycle, GC is fed with commercial feeds and/or several edible types of grass planted, and CMC with commercial feeds, frozen fish, and maize. Additionally, GC and CMC have significantly different farming practices, including stocking density and production. This study focused on more than 15 years of aquaculture ponds in the middle Yangtze River basin, Hubei Province, China. The ponds are located in the center of China and marked on the map (Figure 1). In each pond, sediments collected from 2~3 sites were homogeneously pooled for further analyses of sediment properties, microbial communities, and extracellular enzymatic activities. Six sediment samples from two different local farms were sampled for GC and CMC ponds, respectively, using a Peterson grab sampler (approximately 10 cm depth below sediment) in this study. Each sample was in duplicate: one was immediately kept in cooling boxes in the dark and transplanted to + 4 °C of refrigerated conditions until arriving at the laboratory. The sediments were analyzed for enzyme activities within 2 days. Another was stored in liquid nitrogen and transplanted to −80 °C for microbial analysis.

### 2.2. Sediment Characterization

For each pond, sediment pH was measured using a specialized pH meter (Testo 205, Testo, Germany) by directly inserting the electrodes of the pH meter into the sediment. A 10~30 g wet sample was dried at 105 °C for 24 h to determine dry weight. All pond sediments before nutrient analysis were dried by freeze-drying (Alpha 2-4LD plus; Christ, Osterode am Harz, Germany). Total carbon (TC) was determined with a C:N auto-analyzer. For total nitrogen (TN) determination, samples were calculated based on crude protein content according to the AOAC official crude protein analysis method #2001.11 [19]. Briefly, each sample was weighed and digested in a sample tube using concentrated sulfuric acid at 400 °C for 2 h, and subsequently measured using an automated Kjeldahl analyzer (VELP Scientifica, Usmate Velate, Italy). The conversion factor used for total N estimation was 6.25 for sediment samples. For total phosphorus (TP), samples were weighed and digested in nitric acid, and then the concentrations were detected using a UV spectrophotometer (Model 752, Shanghai Modern Science Co. Ltd., Shanghai, China) with a standard curve of monobasic potassium phosphate solutions.

### 2.3. Sediment Extracellular Enzymatic Activities

Total extracellular enzymatic activities, including leucine aminopeptidase (LAP), β-1,4-glucosidase (BG), and alkaline phosphatase (AP) activities of sediments, were detected according to the multi-concentration method of Hoppe (1983) [20]. Fluorogenic methylumbelliferyl (MUF)-derived compound, MUF-β-glucopyranoside (Sigma-Aldrich), was used as substrate for activity analysis of BG and l-leucine-4 methyl-7-coumarinylamide (Leu-MCA, Sigma-Aldrich) for LAP. The analysis of AP was based on the released p-Nitrophenol (p-NP) from the substrate, p-Nitrophenyl disodium orthophosphate (p-NPP). The hydrolytic enzyme activities were assessed by detecting fluorescence released by hydrolysis of the substrates into the highly fluorescent products MUF at 445 nm and MCA at 440 nm using a spectrofluorometer (Model 960, Shanghai Sanco Co. Ltd., Shanghai, China), and the absorbance of p-NP at 410 nm using a UV spectrophotometer (Model 752, Shanghai Modern Science Co. Ltd.).

### 2.4. Sediment DNA Extraction, 16S rRNA Gene Amplification

Total genomic DNA from 0.3 g of sediment for each pond was extracted using a PowerSoil DNA Kit (Qiagen, Hilden, NRW, Germany) according to the manufacturer’s instructions. The extracted DNA was assessed with 1% agarose gels, and the DNA quality was assessed by Nanodrop 2000. The V3-V4 region of the 16S rRNA gene was amplified using primers 338F (5′- ACTCCTACGGGAGGCAGCA-3′) and 806R (5′-GGACTACHVGGGTWTCTAAT-3′) combined with adapter sequences and barcode sequences. All PCR reactions were performed in a total volume of 50 μL containing 10 μL buffer, 0.2 μL Q5 High-Fidelity DNA Polymerase, 10 μL High GC Enhancer, 1 μL dNTP mixture, 10 μM of each primer, and 60 ng of the extracted DNA. The PCR program was: 98 °C for 2 min, followed by 20 cycles at 98 °C for 30 s, 50 °C for 30 s, 72 °C for 1 min, with a final extension at 72 °C for 5 min. The second-round PCR reactions were performed with a 40 μL volume including 20 μL 2× Phμsion HF MM, 8 μL ddH2O, 10 μM of each primer, and 10 μL PCR products from the first step at the same thermal cycling conditions with 10 cycles. Finally, all PCR products were quantified and pooled together. Subsequently, a sequencing library was generated, and the quality was assessed. Lastly, the library was sequenced on an Illumina HiSeq2500 platform with a 250 bp paired-end sequencing strategy at Biomarker Technologies Corporation (Beijing, China).

### 2.5. Sequencing Data Analysis

Raw tags were obtained using FLASH (v1.2.7) [21]. Raw sequence data were processed in QIIME (v1.8.0) according to a quality-controlled process [22] to obtain high-quality clean tags. The quality filtering and ambiguous base removal were conducted, and the sequence reads were assigned to corresponding samples based on their unique barcode paired-end reads. Chimera sequences were removed using the UCHIME algorithm [23]. The processed sequences with ≥97% similarity were clustered into the same Operational Taxonimic Units (OTUs) by the UCLUST algorithm. Based on previous recommendations, OTUs representing <0.005% of the total number of sequences were discarded for further taxonomic analysis [24]. Taxonomic classifications of each OTU-representative sequence were performed with the MOTHUR program using the SILVA database with 80% confidence threshold. MOTHUR software (v1.30.0) was used to perform alpha diversity analyses, including community richness estimates (Chao and ACE) and community diversity indexes (Shannon and PD). Beta diversity analyses including UPGMA clustering trees were calculated based on Jaccard and Bray–Curtis distances at the OTU levels.

### 2.6. Statistical Analysis

Significant differences in sediment bacterial alpha diversity, sediment properties, and enzyme activities between GC and CMC ponds were determined using a Student’s t-test or Mann–Whitney rank sum test in this study after analysis of the homogeneity of data. The correlations between alpha diversity and sediment biochemical variables and between the enzyme activity and sediment variables in aquaculture ponds were calculated using Pearson correlation coefficients. These analyses were performed using SPSS 21.0 for Windows. Differences in beta diversity of bacterial communities were calculated using permutational multivariate analysis of variance (PERMANOVA) tests by R package software. Redundancy analysis (RDA) was used to calculate the relationship between the sediment biochemical variables (sediment pH, moisture, TC, TN, TP, and C:N) and the sediment bacterial community at the phylum levels. Further, the environmental variables significantly correlated with variations of the bacterial communities were analyzed using the Mantel test, and the relationship between sediment variables and samples was visualized in the plot. Results with *p <* 0.05 between groups were considered statistically significant.

## 3. Results

### 3.1. General Characteristics and Nutrients in Pond Sediments

Physio-chemical properties were analyzed in sediments collected from GC and CMC ponds (Table 1). There were no differences in sediment temperature. However, the sediment pH was lower in GC ponds than in CMC ponds (*p* < 0.001). The moisture content across the ponds ranged from 43.77% to 63.57% of wet sediments, with significant differences in the two types of ponds (*p* < 0.05). The contents of TC, TN, and TP in sediments were significantly different between GC and CMC ponds. The TC content in dry sediments was 21.35 g kg^−1^ in GC ponds and 27.46 g kg^−1^ in CMC ponds (*p* < 0.001). TN and TP contents were 2.49 and 1.14 g kg^−1^ in GC ponds, and 2.98 and 1.62 g kg^−1^ in CMC ponds, respectively (*p* < 0.01 for TN and *p* < 0.05 for TP). No significant differences were observed in ratios of C:N between GC and CMC ponds (*p* > 0.05).

### 3.2. Sediment Enzymatic Activities

**Figure 2:**
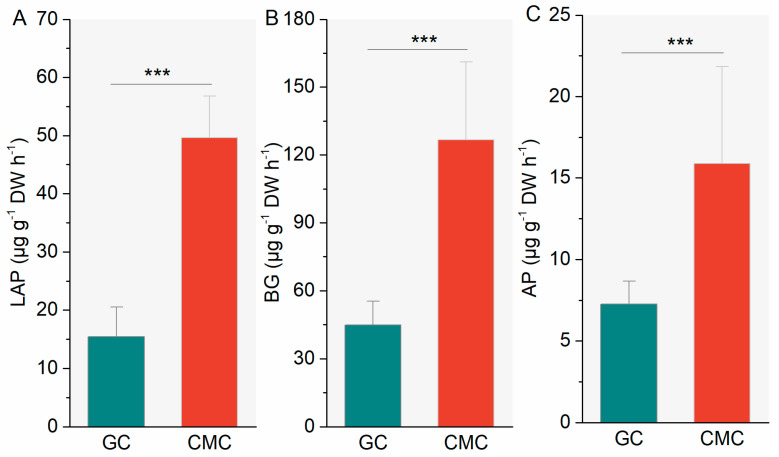
Sediment hydrolytic enzyme activities in ponds with different aquaculture practices. Values are mean (*n* = 6) with standard deviation. (**B**) BG: β-glucosidase; (**A**) LAP: leucine aminopeptidase; and (**C**) AP: alkaline phosphatase. See Table 1 and Figure 1 for abbreviations. *** *p <* 0.001.

When averaged across all the ponds for aquaculture practices, the potential activities g^−1^ in dry sediment of the three enzymes, BG, LAP, and AP, were significantly different between GC and CMC ponds (Figure 2). Sediment BG, LPA, and AP activities were higher in CMC ponds than in GC ponds by 182%, 220%, and 119%, respectively (all *p <* 0.001).

### 3.3. Sediment Bacterial Richness and Diversity

**Figure 3:**
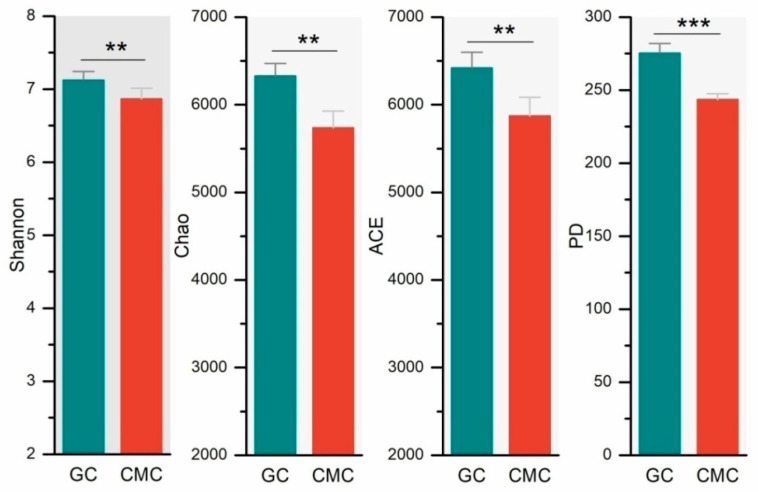
Bacterial richness and diversity for the sediments collected from the ponds with different aquaculture practices. See Figure 1 for abbreviations. ** *p <* 0.01; *** *p <* 0.001.

A total of 848,821 sequences were obtained across all sediment samples, with sequence numbers varying from 68,775 to 72,615 per sample (mean = 70,735). Four indices of bacterial communities were calculated to assess the richness and diversity of sediments (Figure 3). We found a higher Shannon diversity index in GC ponds than CMC ponds (7.12 vs. 6.89, *p* < 0.01). Compared to CMC ponds, GC ponds had the higher bacterial phylogenetic diversity (PD) (275.2 vs. 243.5, *p* < 0.001). In all aquaculture ponds, the numbers of sediment bacterial OTUs varied between 4113 and 5013. GC ponds had significantly more OTUs than CMC ponds (4820 vs. 4185, *p* < 0.01). Similarly, the Chao1 richness estimator of sediments collected from GC ponds was higher than that from CMC ponds (6329 vs. 5736, *p* < 0.01).

### 3.4. Sediment Bacterial Community Structure and Composition

**Figure 4:**
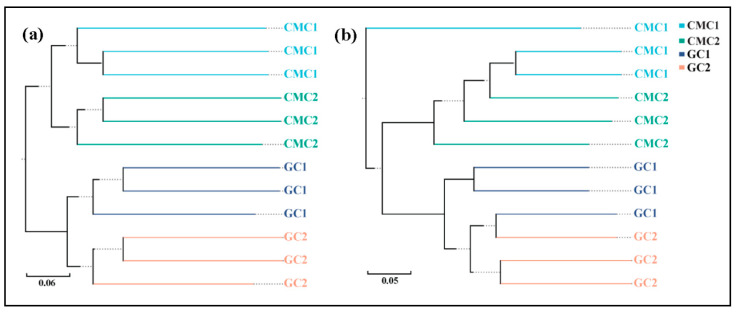
UPGMA clustering of sediment bacterial communities in aquaculture ponds based on (**a**) Jaccard and (**b**) Bray–Curtis distances at the OTU levels. See Figure 1 for abbreviations. The same labels represent sediment samples of the ponds from each site.

To investigate the dependence of sediment bacterial community structure on aquaculture ponds, UPGMA clustering was used to visualize the Jaccard and Bray–Curtis dissimilarities of sediment bacterial communities at the OTU levels between GC and CMC ponds (Figure 4), and then to display clear separations of sediment samples from the two types of aquaculture ponds. The observation was further confirmed using PERMANOVA tests based on the two dissimilarity distance metrics (both *p* < 0.001). The results showed that the pond sediment community structure was significantly affected by farming practices.

**Figure 5:**
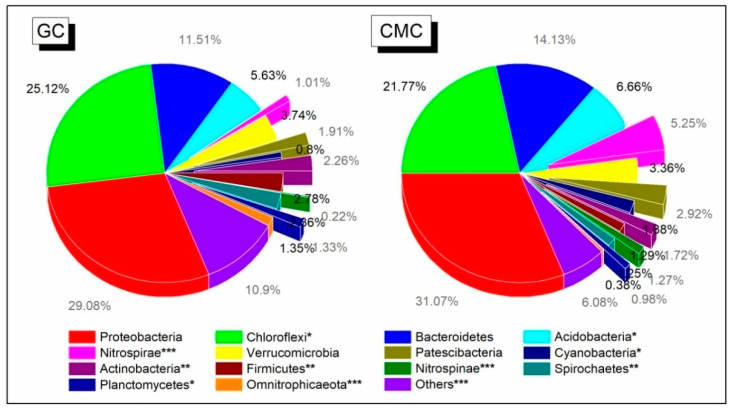
Relative abundance of the dominant bacterial phyla for pond sediments separately according to aquaculture practices. See Figure 1 for abbreviations. * *p* < 0.05; ** *p* < 0.01; *** *p* < 0.001.

Among the bacterial taxa, the dominant OTUs across all sediment samples were classified into 12 phyla (with relative abundance > 1%). Of them, the most abundant taxa were *Proteobacteria*, *Chloroflexi*, *Bacteroidetes*, *Acidobacteria*, *Verrucomicrobia*, *Nitrospirae*, *Patescibacteria*, *Firmicutes*, and *Actinobacteria*, with averaged abundances of 30.3%, 23.7%, 12.9%, 6.2%, 3.6%, 3.1%, 2.4%, 2.1%, and 2.0%, respectively. However, most of the abundant phyla between GC and CMC ponds showed significant compositional differences (Figure 5). *Chloroflexi* (25.1% vs. 21.8%), *Actinobacteria* (5.6% vs. 6.7%), *Firmicutes* (2.8% vs. 1.3%), *Spirochaetes* (2.4% vs. 1.3%), *Planctomycetes* (1.4% vs. 1.0%), and *Omnitrophicaeota* (1.4% vs. 0.4%) had significantly higher abundant proportions in GC ponds than CMC ponds; meanwhile, the relative abundance of *Acidobacteria* (5.7% vs. 6.7%), *Nitrospirae* (1.0% vs. 5.3%), *Cyanobacteria* (0.8% vs. 1.9%), and *Nitrospinae* (0.2% vs. 1.3%) was lower in GC ponds.

### 3.5. Key Factors Affecting Sediment Enzyme Activities and Bacterial Communities

**Figure 6:**
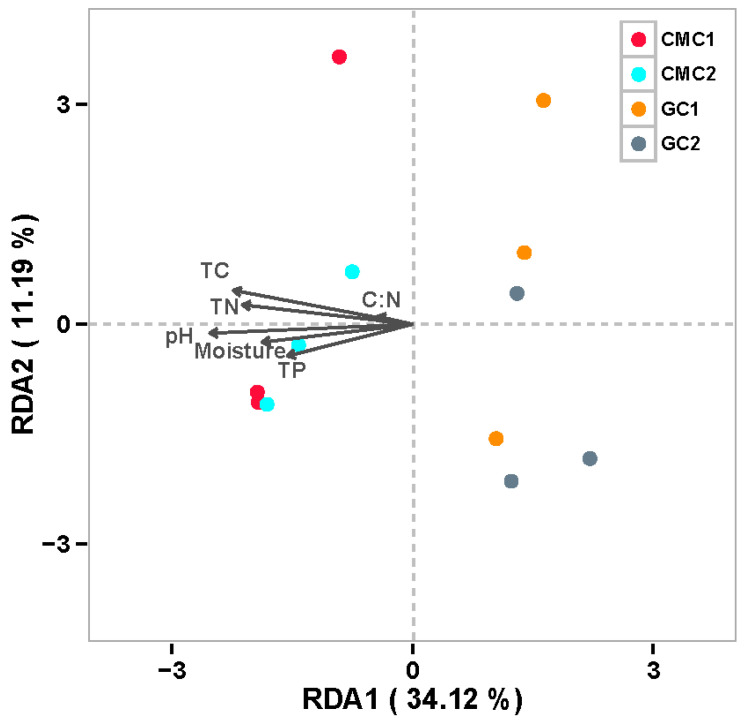
Redundancy analysis of environmental variables for the sediment bacterial communities at the phylum levels in aquaculture ponds. The explanatory variables are shown by different arrows. Filled circles represent sediment samples collected from aquaculture ponds. See Table 1 and Figure 1 for abbreviations.

The factors explaining variations of sediment enzyme activities and bacterial communities among ponds were evaluated by Pearson’s correlation analysis and Redundancy Analysis (RDA). The results showed that LAP and AP positively correlated with all the selected environmental variables (temperature, pH, moisture, TC, TN, and TP) except for C:N ratios (Table 2). BG had significant positive correlations with sediment pH, moisture, TC, and TN. In addition, the correlation analysis indicated that bacterial richness (Chao1 and OTUs) and diversity (Shannon diversity and PD) had negative correlations with sediment pH (all *p <* 0.01) and the concentrations of TC (all *p <* 0.05). Bacterial diversity was negatively correlated with the concentrations of moisture and TN (all *p <* 0.05), while bacterial richness and diversity had no significant correlations with TP and C:N (Table S2). The relationships between environmental variables and bacterial communities at the phylum levels were visualized in an RDA ordination plot, and the variables in the first two axes collectively explained 45.31% of the variance for the bacterial communities among the samples (Figure 6). The differences in the communities between GC and CMC ponds were mainly linked with sediment pH, TC, and TN. Further, based on Euclidean distances, Mantel tests indicated that bacterial communities were mostly affected by sediment pH (r = 0.51, *p* = 0.003), followed by TC (r = 0.47, *p <* 0.002), and TN (r = 0.27, *p* = 0.034).

## 4. Discussion

Fish farming is a very common practice for the agriculture sector; however, as found worldwide, it greatly affects environmental conditions in pond sediments [5,25]. Not surprisingly, high nutrient accumulations in pond sediments are associated with commercial feed input and a low transfer ratio of nutrient output in aquaculture [4,5], which cause diverse biological activities in the sediments. Herein, the present study showed significant differences in sediment nutrient storages, bacterial communities, and enzyme activities between GC and CMC ponds, providing direct evidence that fish and crab farming results in different effects on pond sediments.

The higher concentrations of TC, TN, and TP in CMC pond sediments may reflect more nutrient accumulations deriving from crab farming. As illustrated by the significantly high feed conversion ratios of CMC calculated by Wang et al. (2016) [26] using an integrated analysis, they are generally over twice the ratio of fish dominated by GC [27]. This can be largely attributed to different feeding habits and practices: grass carp swallowing feeds with fewer feed wastes compared to crab biting with feeds. In addition, GC ponds usually stocked with some filter-feeding species, such as bighead carp (*Hypophthalmichthys nobilis*), to consume phytoplankton can utilize excess nutrients for maintaining water quality. In most previous studies, the amounts of nutrient loadings in the environment have been theoretically calculated and were different for aquaculture species [28]. Our nutrient determinations provide direct evidence of different nutrient loadings in the ponds. It is supposed that such differences in aquaculture practices can raise some issues concerning the biological effects of sediment nutrient cycling and utilization and the associated environmental impacts.

The settling materials are mainly composed of labile feed residues and fish feces favoring benthic microbial growth and metabolism [8]. It has been reported that fish farming affects microbial compositions of marine sediments due to the production of waste materials [17,29]. In this study, we found that this is also the case for inland aquaculture ponds. Although the majority of abundant sediment-dwelling bacteria were the same in GC and CMC ponds, significant differences in the sediment communities were observed. In the sediments, several bacterial phyla, including *Proteobacteria*, *Chloroflexi*, *Bacteroidetes*, and *Acidobacteria*, were the common species [30,31], and the bacterial communities are closely associated with sediment nutrient deposition from feed input [31]. Sequencing analysis revealed that most abundant bacterial taxa had phylum-level changes in GC and CMC ponds. Different sediment factors contribute to significant differences in the bacterial communities. For instance, TC and TN were positively correlated with the abundance of *Nitrospirae*, which was composed of a group of bacteria able to utilize N [32]. Concerning bacterial alpha diversity, there were remarkably negative correlations with the sediment TC content, as well as TN and TP contents, as revealed by the decline in the richness and diversity with N and P input in a previous study [33]. The observations are in accordance with the classical ecological view that immoderate nutrient availability could result in decreased species diversity in an ecosystem. In most circumstances, it is accepted that the loss of species diversity may threaten the ecosystem stability, health, and interactions due to excessive nutrient accumulations. In aquaculture, earthen ponds have a demonstrated ability to trap nutrients and generally enrich higher nutrient concentrations compared to other sediment/soil environments, such as lakes, rivers, and forests, due to continuous aquaculture practices for decades. Correspondingly, shifts in the bacterial communities may be a function of changes in sediment nutrient decomposition and utilization.

Similarly, environmental factors, such as pH and moisture, are powerful controllers for bacterial growth and contributing to ecosystem processes [34,35,36,37]. Unexpectedly, despite such small differences in sediment pH (by up to about 0.4 units) within the neutral range between GC and CMC ponds, it did account for a significant proportion of variabilities associated with the observed changes in phylogenetic diversity and community structure. Across the corresponding pH gradient, these patterns were also observable in soils at the continental scale, although the neutral range showed a relatively small change [38], suggesting bacterial communities influenced by pH probably due to some taxa having relatively narrow growth tolerances. However, whether sediment pH across the wide gradient, such as soil pH, is a universal predictor of bacterial community structure needs further studies in aquaculture ponds [38,39]. In aquaculture ponds, the sediments are generally anoxic. Other factors, such as the redox potential, may also be largely associated with sediment microbial communities, including Archaea. This study only focuses on bacteria; meanwhile, Archaea could be present in these systems. We will further study the relationship between the redox potential and the microbiota, as well as the responses of microbiota to these factors to better understand sediment microbial assembly.

Extracellular enzymes can generally be used as the proximate agents of organic matter decomposition, and the enzyme activities are useful proxy measures of substrate nutrient cycling. However, the concept proposed and/or the findings confirmed by researchers are mainly based on various terrestrial ecosystems [18]. For hydrolases, a meta-analysis of collecting samples along the hydrologic gradient from terrestrial soil, to lentic wetland sediment, and to lotic river sediment revealed approximate ratios of 1:1:1 for specific C (BG), N (LAP and β-1,4-N-acetylglucosaminidase, NAG), and P (AP) acquisition activities; however, the site variability within/between ecosystems was extremely large [40], indicating the heterogeneous properties of soil and/or sediment environments. The activities of BG, NAG, and AP, but LAP g^−1^ soil, increased with organic matter concentrations among the terrestrial soils. In this study, sediment BG and AP activities in ponds showed statistically significant relationships to TC in univariate models with strong positive trends; LAP activities also had a positive relationship with TC contents. It is difficult to calculate whether the distribution of specific C, N, and P enzymatic activities converges on 1:1:1 due to no available data on NAG activity in this study. Despite great differences in BG and AP activities between GC and CMC ponds, their ratios of BG:AP were consistently convergent, further suggesting that enzymatic potentials for hydrolysing organic materials are associated with specific substrate chemistry and availability among the ecosystems. However, there are potential limitations of only measuring a subset of enzymes to relate to sediment nutrient utilization. In further studies, we will conduct comprehensive investigations of enzymatic activities associated with different categories of nutrients in the pond sediments. In ecosystems, greater acquisition activities of enzymes mean relatively lower elemental availability of carbon, nitrogen, and phosphorus [41]. Unexpectedly, positive relationships between sediment BG, LAP, and AP enzymatic activities and the corresponding nutrients were observed in pond sediments; however, this contradicts the results obtained from terrestrial soils [42,43] and questions the proposed enzymatic method to infer nutrient limitations for microbes in environmental samples [18,40]. Compared to terrestrial ecosystems, a small sample size in pond ecosystems may make this observation uncertain, but this study potentially proposes concerns of grow-limitation steps of sediment microbes and their relationships with enzymatic activities in aquaculture ponds. Notably, the pond ecosystems that are heavily disturbed are significantly dependent on specific farming practices. Therefore, future studies on sediment extracellular enzyme activities in aquatic ecosystems are needed to collect data from various aquaculture pond ecosystems to elucidate the underlying mechanisms driving the responses of extracellular enzyme activities to detailed nutrient storages in sediments.

## 5. Conclusions

Our results indicate that farming practices of fish and crab have significantly different effects on the bacterial community and enzymatic activity in sediments of aquaculture ponds. In this study, a lack of control ponds led to limitations in understanding the long-terms changes in the sediment bacterial communities; however, this study provides insights into sediment biological data in different ponds. The major contribution to the differences in fish and crab ponds may derive from their different farming practices, which induce different amounts of deposition of uneaten feeds and fecal matter, and microbial assimilations. The sediment properties, especially pH and TC, showed significant positive relationships with sediment hydrolytic enzyme activities, and they were also associated with the variations in the bacterial community. More specific studies regarding stoichiometric patterns of sediment enzyme activities, combined with various farming practices, are needed to elucidate the mechanisms behind the differences in pond ecosystems.

## Figures and Tables

**Table 1:** Characterizations of sediments from aquaculture ponds examined for this study.

Ponds	pH	Moisture (%)	TC (g kg^−1^)	TN (g kg^−1^)	TP (g kg^−1^)	C:N
GC	7.19 ± 0.11	52.56 ± 5.45	21.35 ± 1.53	2.49 ± 0.31	1.14 ± 0.35	8.97 ± 0.89
CMC	7.55 ± 0.13	59.87 ± 2.61	27.46 ± 1.90	2.98 ± 0.20	1.62 ± 0.37	9.24 ± 0.71
*p*	***	*	***	**	*	ns

*** *p* < 0.001 ** *p* < 0.01 * *p* < 0.05, ns: No significant differences.

**Table 2:** Pearson’s correlation coefficients of sediment enzyme activities on the sediment properties in aquaculture ponds.

Enzymatic Indices	pH	Moisture	TC	TN	C:N	TP
LAP	0.874 **	0.769 **	0.803 **	0.788 **	0.141	0.629 *
BG	0.696 *	0.616 *	0.774 **	0.683 *	0.223	0.429
AP	0.623 **	0.691 *	0.826 **	0.650 *	0.338	0.620 *

** *p* < 0.01 * *p* < 0.05.

## Data Availability

The sequences in this study are available under NCBI’s BioProject accession PRJNA612144.

## Supplementary Materials

The following are available online at https://www.mdpi.com/2076-2607/9/3/501/s1, Table S1: Water quality parameters of aquaculture ponds examined for this study. Table S2: Pearson’s correlation coefficients of sediment bacterial richness and diversity on the sediment properties in aquaculture ponds.
