# Supplementary material for: Bacterial Communities and Enzymatic Activities in Sediments of Long-Term Fish and Crab Aquaculture Ponds
Source: Microorganisms. 2021 Feb 26;9(3):501. doi: 10.3390/microorganisms9030501 (PMC7996777; doi:10.3390/microorganisms9030501)
Supplement: Supplementary file 1 [file microorganisms-09-00501-s001.pdf]

Supplementary material

Bacterial communities and enzymatic activities in sediments of long-term fish and crab aquaculture ponds

Zhimin Zhang[1], Qinghui Deng[1,2], Lingling Wan[1,2], Xiuyun Cao[1], Yiyong Zhou[1], Chunlei Song[1,*]

[1] *Key Laboratory of Algal Biology, State Key Laboratory of Freshwater Ecology and Biotechnology, Institute of Hydrobiology, Chinese Academy of Sciences, Wuhan 430072, P. R. China*

[2] *University of Chinese Academy of Sciences, Beijing 100039, P. R. China*

*Corresponding author: Dr. Chunlei Song; E-mail address: clsong@ihb.ac.cn

Table S1 Water quality parameters of aquaculture ponds examined for this study.

Ponds	T (℃)	DO (mg)	C (us/cm)	TDS (mg/L)	SAL (ppt)	pH	OPR	Depth (m)
GC	6.14	13.10	319.80	325.43	0.24	8.93	-146.07	2.58
	0.17	1.55	28.38	29.99	0.02	0.19	166.93	0.23
CMC	6.37	12.45	252.08	227.07	0.18	8.90	-113.25	0.93
	0.16	0.76	18.35	43.76	0.02	0.30	237.28	0.21

Note. GC, Grass carp; CMC, Chinese mitten crab.

Table S2 Pearson's correlation coefficients of sediment bacterial richness and diversity on the sediment properties in aquaculture ponds.

Enzymatic indices	pH	Moisture	TC	TN	C:N	TP
ACE	-0.797**	-0.546	-0.706*	-0.480	-0.336	-0.497
Chao	-0.769**	-0.564	-0.741**	-0.501	-0.357	-0.49
Shannon	-0.895***	-0.669*	-0.608*	-0.592*	-0.014	-0.538
PD	-0.895***	-0.669*	-0.706*	-0.609*	-0.175	0.503
